# Early Esophageal Perforation Following Anterior Cervical Discectomy and Fusion Treated With Controlled Esophageal Fistula and TachoSil® Coverage: A Case Report

**DOI:** 10.7759/cureus.68591

**Published:** 2024-09-03

**Authors:** Abdulrahman H Alashkar, Nagi A Massoud, Feras Al-Rawashdeh, Mohammad A Aljawash

**Affiliations:** 1 Department of Surgery, Dr. Sulaiman Al-Habib Medical Group, Buraidah, SAU

**Keywords:** glycopyrrolate, esophagocutaneous fistula, tachosil®, esophageal injury, anterior cervical discectomy

## Abstract

Anterior cervical discectomy and fusion (ACDF) is a commonly performed procedure. One of its most feared complications is esophageal injury (EI). There is no standard approach on how to treat these injuries. TachoSil^®^ (Corza Medical GmbH, Düsseldorf, Germany) is a fibrinogen-containing patch that has been used in various surgical areas. Here, we present a 68-year-old male patient who was diagnosed with an EI with secondary surgical site infection following a three-level ACDF (C4/5, C5/6, and C6/7). Initially, the patient underwent incision and drainage (I&D) of the surgical site abscess, primary repair of the esophageal tear, and esophageal catheter placement to create a controlled esophagocutaneous fistula. Postoperatively, he was diagnosed with a leak and underwent a second I&D procedure. The primary repair of the EI was augmented with a TachoSil patch, and the patient was started on glycopyrrolate. The site of EI was well-sealed with no re-leaks, and the patient was discharged after he had completed a course of intravenous (IV) antibiotics and had been on parenteral nutrition for a total of 40 days. This case shows that the use of TachoSil to augment the repair of ACDF-associated EI, and glycopyrrolate to decrease salivation could decrease the risk of leak and enhance the healing process. This is an observation that needs to be scrutinized in future studies.

## Introduction

Anterior cervical discectomy and fusion (ACDF) is a commonly performed procedure. It’s generally safe, but one of its most serious and feared complications is EI. There is no standard approach on how to treat these injuries, largely because of their rarity. Also, their treatment is often lengthy, involves multiple surgical procedures, and is often made even more complicated by the presence of implant materials and secondary infections [1,2]. TachoSil® (Corza Medical GmbH, Düsseldorf, Germany) is a fibrinogen-containing patch that has been used as a sealant and a hemostatic agent in various surgical areas, and glycopyrrolate is an anticholinergic medication that inhibits salivation. Here, we present a case of ACDF-associated EI in which TachoSil and glycopyrrolate were used in the course of its management.

## Case presentation

A 68-year-old male patient, a known case of hypertension, presented to the emergency department with fever, dysphagia, and fatigue. Five days prior, he had a plated three-level ACDF (C4/5, C5/6, and C6/7). He had been discharged on the third postoperative day. However, his symptoms developed quickly over about 24 hours prior to his presentation.

On physical examination, the patient was fully conscious, yet ill-looking. He had a high-grade fever (40^o^ Celsius), but his other vital signs were within normal range. His surgical site was dry, with no wound discharge or appreciable swelling. Further examination did not reveal additional findings. His initial blood work showed high levels of serum inflammatory markers and venous lactate as shown in Table 1.

**Table 1:** Inflammatory markers and venous lactate levels at the time of the patient's presentation.

Test	Test result	Reference range
White blood cell count	21,000 cells/μL	4,000-11,000 cells/μL
C-reactive protein	205 mg/L	˂ 5 mg/L
Pro-calcitonin	0.84 ng/ml	˂ 0.1 ng/ml
Venous lactate	2.24 mmol/L	0.5-2.2 mmol/L

**Figure 1:**
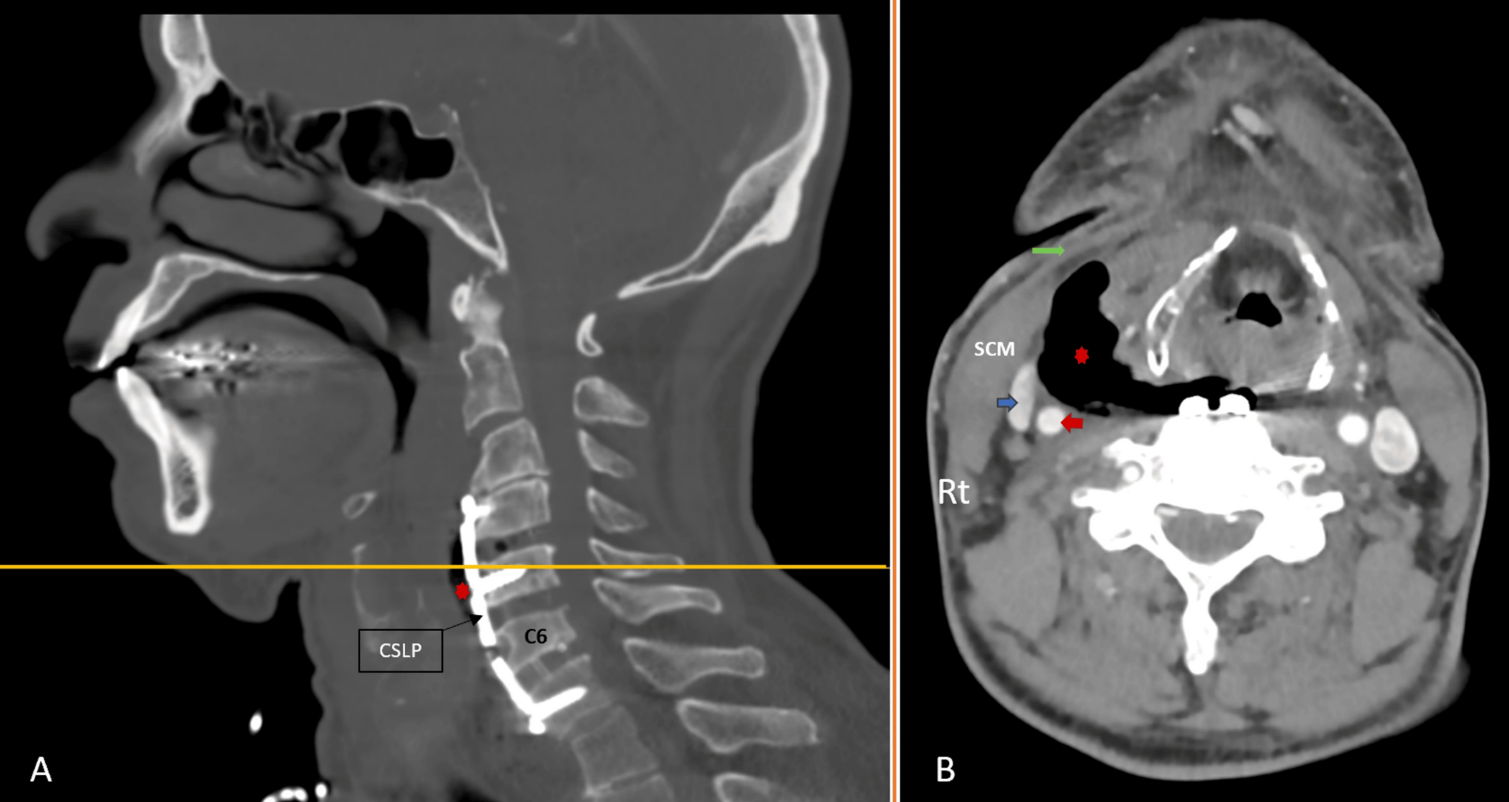
Neck CT with IV contrast taken upon the patient’s presentation with dysphagia and fever (A) Sagittal view showing the surgical fusion with a cervical spine locking plate (CSLP), and screws. Note the air at the prevertebral area (red star). (B) Axial view taken at the level of the fifth cervical vertebra (the yellow line in (A)). It shows the presence of an abscess evident by the presence of air (red star), and air-fluid level at the base. Notice that the air/abscess is present within the surgical plane: below the platysma (green arrow), and medial to the sternocleidomastoid muscle (SCM). The red and blue arrows are pointing at the common carotid artery, and the internal jugular vein, respectively.

Computed tomography (CT) of the neck with contrast showed well-placed implants, and right paratracheal collection (Figure 1).

The patient was kept on broad-spectrum IV antibiotics (meropenem 1 g every eight hours and vancomycin 1 g every 12 hours) and underwent surgical incision and drainage (I&D) of the abscess. Intraoperatively, a collection of pus with food particles was found under the platysma, which was sampled and drained. Also, a 5 mm full-thickness right posterolateral esophageal tear was found at about the level of the seventh cervical vertebra. The area was thoroughly washed with saline mixed with 10% povidone. A nasogastric tube (NGT) was placed. Then, a catheter (Foley, rubber, size 12F) was inserted at the esophageal perforation site (positioned distally), and we performed a full-thickness (single layer) repair of the tear using a polydioxanone suture (PDS). Finally, a surgical drain was placed alongside the esophagus. Culturing of the intraoperative samples grew *Klebsiella pneumoniae*, and the antimicrobial therapy was changed from meropenem and vancomycin to ceftriaxone 2 g IV one every 24 hours (as per the sensitivity testing) and metronidazole 500n mg IV every eight hours (as an empirical anti-anaerobic therapy).

**Figure 2:**
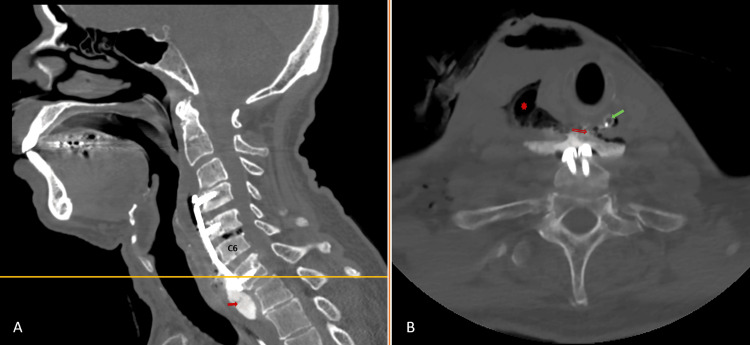
Neck CT with oral Gastrografin taken when the output of the surgical drain was increasing following the first incision and drainage procedure. (A) Sagittal view showing the contrast leak (red arrow). (B) Axial view taken at the level of the seventh cervical vertebra (the yellow line in (A)), where the site of esophageal injury/leak is present (red arrow). The green arrow is pointing at the nasogastric tube.

The patient was improving as evidenced by his overall sense of well-being, absence of fever, stable vital signs, and decreasing inflammatory markers. Nevertheless, on the seventh day following the I&D, the output of the surgical drain increased over time with a saliva-like output (whitish, frothy, nonfoul smelling). On the 10^th^ day following the I&D, a repeat CT scan with oral Gastrografin revealed a re-collection with a leak from the esophageal tear site (Figure 2). So, another I&D was performed. Intraoperatively, a non-purulent, nonfoul-smelling collection of saliva was noted with good granulation tissue at the surgical bed. Samples were taken, irrigation was done, and the site of the esophageal tear was augmented with a TachoSil patch. Following the second drainage, the patient was started on parental nutrition. Also, he was started on IV glycopyrrolate (0.2 mg every 12 hours). 

The samples taken from the second I&D grew *Staphylococcus epidermidis*, and the antimicrobial therapy was adjusted accordingly. The output of the surgical drain became serosanguinous and was decreasing gradually. After 20 days from the second I&D (30 days from the first I&D), the patient was clinically stable, the surgical drain was yielding minimal serosanguinous output, and the level of inflammatory markers (WBC and C-reactive protein (CRP)) normalized. So, the antimicrobial therapy was discontinued. 

**Figure 3:**
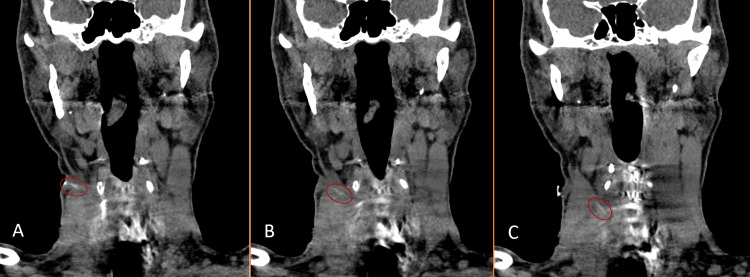
Neck CT with oral Gastrografin conforming the formation of an esophagocutaneous fistula and the absence of leak. (A-C) Sequential coronal views showing the presence of contrast (faint radio-dense line) in the esophagocutaneous fistula, which is indicated by the red circles.

The esophageal catheter was removed one month following the second I&D, and the patient was restarted on NGT feeding (alongside parenteral nutrition) for 10 days. Then, the patient was allowed to start oral intake. Two days later, the surgical drain was removed and a final neck CT scan with oral Gastrografin was performed and confirmed the formation of a controlled (esophagocutaneous) fistula with no leak (Figure 3). In total, the patient remained on parenteral nutrition for 40 days.

## Discussion

The anterior approach to the cervical spine is a commonly performed procedure that was devised back in 1958 by Smith and Robinson [3] and Cloward [4]. It’s generally safe, and the occurrence of serious complications is infrequent. One such complication is EI, which has an incidence of around 1%; a range of 0.02-1.6% has been reported [5,6].

Diagnosing and managing this surgical entity is exceedingly difficult due to several factors, including its rarity, the presence of implant materials, the contaminated nature of the esophageal lumen, and the frequent presence of coexisting infections. Moreover, its most common presenting symptom is dysphagia, which is also a common complication for any patient undergoing ACDF, with some studies reporting it to be the most common complication following ACDF [1,7]. In our patient, the presentation was more dramatic with worsening dysphagia and high-grade fever, which made suspecting an EI clinically easier.

The most common cause of ACDF-associated EI has been reported in a systematic review to be hardware failure (41%) [1]. In the current case, however, the likely cause was an intraoperative injury, which explains the patient’s early presentation on day 6 postoperatively.

ACDF-associated EI has been categorized into early and late based on the time of diagnosis being earlier or later than 30 days, respectively. This distinction has been made mainly based on the likely mechanism of injury, and a different management approach has been proposed for each. Early injuries are typically iatrogenic and managed with primary closure with or without flap reconstruction; while late injuries are secondary to hardware failure and managed with primary repair, flap reconstruction, and implant removal with or without posterior fixation [2].

Nevertheless, multiple surgical procedures are often needed, which reflects the complexity of managing this complication. In a review of 173 patients, 34.5% of cases required several (2-10) surgical interventions during the course of treatment [2]. In our case, the patient underwent two surgical interventions. First, an I&D of a surgical site abscess with primary esophageal repair and placement of an esophageal catheter to create a controlled (esophagocutaneous) fistula. Then, due to the development of a re-collection, a second I&D was performed, and the site of EI was augmented with a TachoSil patch.

Using a catheter to generate a controlled fistula is meant to create a low-pressure conduit through which secretions can be drained; thus, decreasing the risk of leak, and subsequently helping the infection to resolve and the injury site to heal. This has been used in esophageal surgery with the catheter being a T-tube [8]. In our case, we used a Foley catheter due to the unavailability of a T-tube. TachoSil and glycopyrrolate have been utilized in this case for the same ultimate goal, which is decreasing the risk of leak to help with infection resolution and injury healing.

TachoSil is a sealant patch that contains human fibrinogen and thrombin. It can be used as a hemostatic agent, or as a sealant. It has been used across surgical fields such as gynecological and colorectal surgery [9,10]. In esophageal surgery, it has been used as a sealant in planned esophagogastric anastomoses, and in iatrogenic perforations following endoscopy procedures [11,12].

Glycopyrrolate is an anticholinergic medication. It has several indications, but it is mainly used to inhibit secretions (salivary and respiratory) or to prevent reflex bradycardia. Its use has been investigated and shown effective in children with anastomotic leaks following primary repair of esophageal atresia, where it facilitated healing and allowed earlier return to enteral feeding [13].

Nevertheless, to the best of our knowledge, the use of neither TachoSil nor glycopyrrolate has been discussed with regard to ACDF-associated EI. With such injuries, the tear is not controlled, the surrounding tissue is frequently infected and there are implant materials. All these factors make the management of ACDF-associated EI more complicated than that of surgical anastomosis leaks or esophageal injuries induced by other mechanisms.

## Conclusions

Esophageal injuries are one of the rare, but serious complications of ACDF. They frequently require lengthy treatment and multiple surgical procedures. TachoSil is a fibrinogen-containing patch with various surgical applications, and glycopyrrolate is an anticholinergic medication. The use of TachoSil in augmenting the site of EI and glycopyrrolate in decreasing salivation could decrease the risk of leak, thus, enhancing the healing process. This is an observation that needs to be scrutinized in future studies.
